# Dimensionality of Social Networks Using Motifs and Eigenvalues

**DOI:** 10.1371/journal.pone.0106052

**Published:** 2014-09-04

**Authors:** Anthony Bonato, David F. Gleich, Myunghwan Kim, Dieter Mitsche, Paweł Prałat, Yanhua Tian, Stephen J. Young

**Affiliations:** 1 Department of Mathematics, Ryerson University, Toronto, Ontario, Canada; 2 Computer Science Department, Purdue University, West Lafayette, Indiana, United States of America; 3 Electrical Engineering Department, Stanford University, Stanford, California, United States of America; 4 Laboratoire J.A. Dieudonné, Université de Nice Sophia-Antipolis, Nice, France; 5 Department of Mathematics and Statistics, York University, Toronto, Ontario, Canada; 6 Mathematics Department, University of Louisville, Louisville, Kentucky, United States of America; Wake Forest School of Medicine, United States of America

## Abstract

We consider the dimensionality of social networks, and develop experiments aimed at predicting that dimension. We find that a social network model with nodes and links sampled from an *m*-dimensional metric space with power-law distributed influence regions best fits samples from real-world networks when *m* scales logarithmically with the number of nodes of the network. This supports a logarithmic dimension hypothesis, and we provide evidence with two different social networks, Facebook and LinkedIn. Further, we employ two different methods for confirming the hypothesis: the first uses the distribution of motif counts, and the second exploits the eigenvalue distribution.

## Introduction

Empirical studies of on-line social networks as undirected graphs suggest these graphs have several intrinsic properties: highly skewed or even power-law degree distributions [1], [2], large local clustering [3], constant [3] or even shrinking diameter with network size [4], densification [4], and localized information flow bottlenecks [5], [6]. These are challenging properties to capture in concise models of social network connections and growth [7]–[9], and many models only possess them in certain parameter regimes. One model that captures these properties *asymptotically* is the geometric protean model (GEO-P) [10]. It differs from other network models [1], [4], [11], [12] because all links in geometric protean networks arise based on an underlying metric space. This metric space mirrors a construction in the social sciences called *Blau space* [13]. In Blau space, agents in the social network correspond to points in a metric space, and the relative position of nodes follows the principle of *homophily* [14]: nodes with similar socio-demographics are closer together in the space.

In order to accurately capture the observed properties of social networks—in particular, constant or shrinking diameters—the dimension of the underlying metric space in the GEO-P model must grow logarithmically with the number of nodes. The logarithmically scaled dimension is a property that occurs frequently with network models that incorporate geometry, such as in multiplicative attribute graphs [7] and random Apollonian networks [15]. Because of its prevalence in these models, the logarithmic relationship between the dimension of the metric space and the number of nodes has been called the *logarithmic dimension hypothesis* [10]. This hypothesis generalizes previous analysis which shows that individuals in a social network can be identified with relatively little information. For instance, Sweeney found that 87% of the U.S. population had reported attributes that likely made them unique using only zip code, gender and date of birth, and concluded that few attributes were needed to uniquely identity a person in the U.S. population [16]. Here, we find evidence of the log-dimension property in real world social networks.

We emphasize that the present paper is the first study that we are aware of which attempts to quantify the dimensionality of social networks and Blau space. While we do not claim to prove conclusively the logarithmic dimension hypothesis for such networks, our experiments, such as those of [16], suggest a much smaller dimension in contrast to the overall size of the networks. Interestingly, speculation on the low dimensionality of social networks arose independently from theoretical analysis of mathematical models of social networks in [7], [10], [15].

Our findings provide evidence for dimensional properties underlying social networks that have a number of potential applications in future studies. First, the dimensional properties could be used for further classification and characterization of different types of networks. Second, many NP-hard optimization problems related to graph properties and community detection are polynomial time solvable in a low dimensional metric space, and thus, our findings suggest new techniques to explore for understanding why we may expect to solve these problems in social networks. Finally, if techniques to find these dimensions emerge, we should be able to create powerful new methods to harness the insight they offer into the network structure.

### MGEO-P

The particular network model we study is a simple variation on the GEO-P model that we name the memoryless geometric protean model (MGEO-P), since it enables us to approximate a GEO-P network without using a costly sampling procedure. Both GEO-P and the MGEO-P model depends on five parameters described in Table 1.

**Table 1:** The parameters of the MGEO-P model.

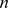	the total number of nodes
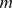	the dimension of the metric space
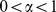	the attachment strength parameter
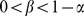	the density parameter
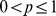	the connection probability

The nodes and edges of the network arise from the following process. Initially the network is empty. At each of 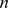 steps, a new node 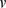 arrives and is assigned both a random position 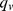 in 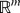 within the unit-hypercube 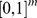 and a random rank 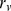 from those unused ranks remaining in the set 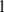 to 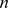. The influence radius of any node is computed based on the formula:
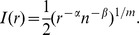



With probability 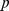, the node 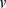 forms an undirected connection to any preexisting node 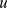 where 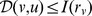, where the distances are computed with respect to the following metric:
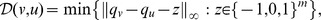



and where 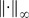 is the infinity-norm. We note that this implies that the geometric space is symmetric in any point as the metric “wraps” around like on a torus. The volume of space influenced by the node is 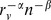. Then the next node arrives and repeats the process until all 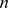 nodes have been placed. In the MGEO-P model, the process ends here, whereas in the GEO-P model, the network then removes the least-recently added node, and inserts a new node following the same procedure. This iterative replacement process continues until it reaches it reaches a random point.

**Figure 1:**
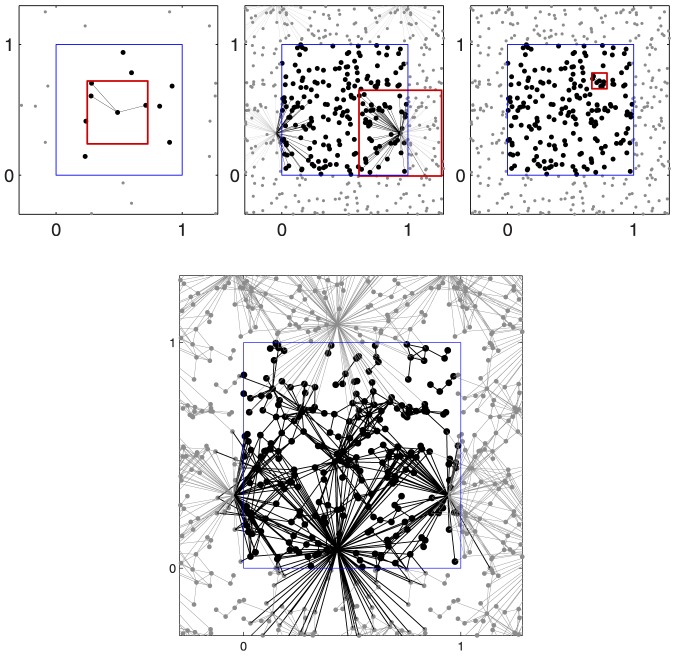
An example describing the MGEO-P process on a graph with 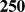 nodes in the unit square with torus metric, where 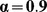 and 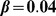 and 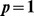. Each figure shows the graph “replicated” in grey on all sides in order to illustrate the torus metric. Links are drawn to the closest replicated neighbor. The blue square indicates the region 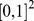. *Top row (left to right)* The MGEO-P process begins with relatively few nodes, and thus, nodes must have large influence radii (red squares) to link anywhere. As more nodes arrive, large radii result in many connections, modeling influential users, and small radii result in a few connections, modeling standard users. *Bottom row* Illustrates the final constructed graph.

Figure 1 illustrates two features of the model. First, after a few steps, only a few nodes exist and even a large influence region will only produce a few links. Second, when the number of steps approaches 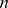, a large influence region will produce many links. The idea behind the model is a simple abstraction of the growth of an on-line social network. When the network is first growing (few steps), even influential members will only know a few other members who have also joined. But after the network has been around for a while (many steps), influential members will begin with many friends.

We formally prove that the MGEO-P model has the following properties. Let 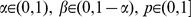 and 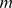 be positive integer. The following statements hold with probability tending to 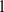 as 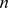 tends to 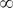. See the MGEO-P section of File S1 for the proofs. We actually show these results hold with extremely high probability, which is a stronger notion that implies probability tending to 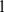.

1. Let 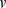 be a node of 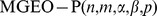 with rank 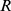 that arrived at step 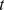. Then
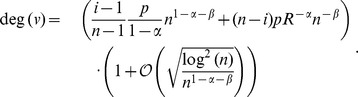



This result implies that the degree distribution follows a powerlaw with exponent 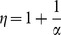.

2. The average degree of node of 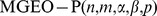 is
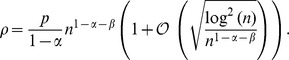



3. The diameter of 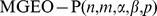 is 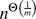.

This last property suggests that, ignoring constants, for a network with 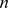 nodes and diameter 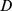, the expected dimension based on the MGEO-P model is
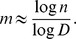



Thus, like some network models that incorporate geometry [7], [15], in the MGEO-P model, the dimension 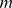 must scale logarithmically in order for the diameter to remain constant as 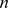 increases.

### Experimental Design and Graph Summaries

**Figure 2:**
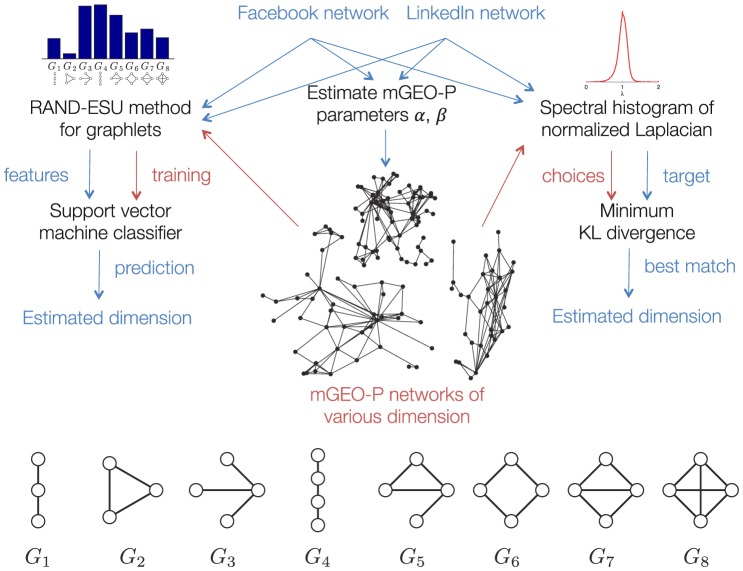
At left and center, we have the steps involved in fitting via graphlets; at right and center, we have the steps involved in fitting via spectral histogram. Throughout, red lines denote the flow of features for the MGEO-P networks whereas blue lines denote flow of features for the original networks. At the bottom, we show an enlarged representation of the 8 graphlets we use.

Both graph motifs and spectral densities are numeric summaries of a graph that abstract the details of a network into a small set of values that are independent of the particular nodes of a network. These summaries have the property that isomorphic graphs have the same values, and we will use these summaries to determine the dimension of the metric space that best matches Facebook and LinkedIn networks as illustrated in Figure 2. Graph motifs, graphlets, or graph moments are the frequency or abundance of specific small subgraphs in a large network. We study undirected, connected subgraphs up to four nodes as our graph motifs (with the exception of the number of edges, or two-node motifs, as the networks are created to preserve this count). This is a set of 8 graphs shown in at the bottom of Figure 2 along with the single two node graph of an edge. The spectral density of a graph is the statistical distribution of eigenvalues of the normalized Laplacian matrix as indicated in the upper right of that figure. These eigenvalues indicate and summarize many network properties including the behavior of a uniform random walk, the number of connected components, an approximate connectivity measure, and many other features [17], [18]. Thus, the spectral density of the normalized Laplacian is a particularly helpful characterization that captures many such separate network properties.

We study dimensional scaling in social networks by comparing samples of the MGEO-P networks of varying dimensions with samples of social network data from Facebook and LinkedIn. We pay particular attention to the relationship between the number of nodes 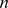 of the network and the dimension 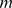 of the best fit MGEO-P network. In order to determine what underlying dimension for MGEO-P best fits a given graph, we employ two distinct methods. For one experiment, we use features known as graph motifs, graphlets, or graph moments in concert with a support vector machine (SVM) classifier. This approach has been used successfully to determine the best generative mechanism of a network [19] and to select parameters of a complicated network models to fit real-world data [9], [20]. In a second experiment, we use spectral densities of the normalized Laplacian matrix of a graph and a Kullback-Leibler divergence (KL divergence) similarity measurement, which has been used to match protein networks between species [21], [22]. We find evidence of the logarithmic dimension hypothesis in both cases.

### The data

**Figure 3:**
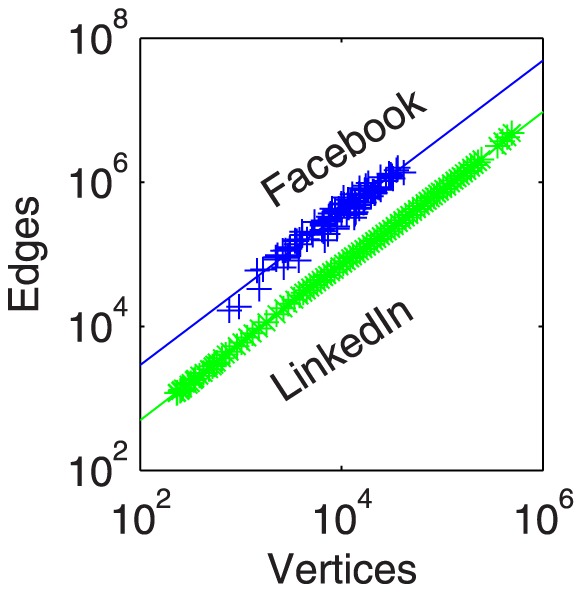
The scale of the network data involved in our study varies over three orders of magnitude. We see similar scaling for both types of networks, but with slightly different offsets. For Facebook, 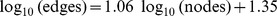 with 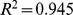; for LinkedIn 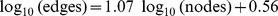 with 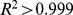. The regularity in the LinkedIn sizes is due to our construction of those networks.

Facebook distributed 100 samples of social networks from universities within the United States measured as of September 2005 [23], which range in size from 700 nodes to 42,000 nodes. We call these networks the Facebook samples. The LinkedIn samples were created from the LinkedIn connection network together with the creation time of each connection from May 2003 to October 2006. To perform our experiments on networks of different size, we build 71 snapshots of the LinkedIn network at various timestamps. We then extracted a dense subset of their graph at various time points that is representative of active users; we used the 5-core of the network for this purpose [24]. The 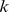-core of a network is a maximum size subset of vertices such that all vertices have degree 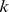. See Figure 3 and the full statistics tables of File S1 for additional properties of these networks. In both networks, the number of edges per node grows at essentially the same rate.

## Results

**Figure 4:**
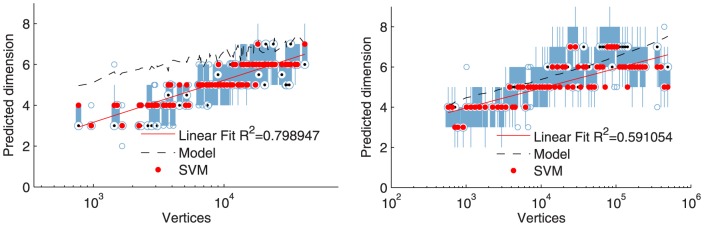
Facebook dimension at left, LinkedIn dimension at right. Each red dot (SVM) is the predicted dimension computed via graphlet features and a support vector machine classifier. For the Facebook data, we find that 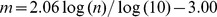. For the LinkedIn data, we find that 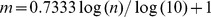. And these are plotted as the red linear fit line. Our theoretical model predicts a dimension of 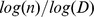 and we plot this as the dashed line. In each figure, we show the variance in the fitted dimension as a box-plot. We estimate the variance by using only 20% of the original training data and repeating over 50 trials. There are only a few outliers for small dimensions.

**Figure 5:**
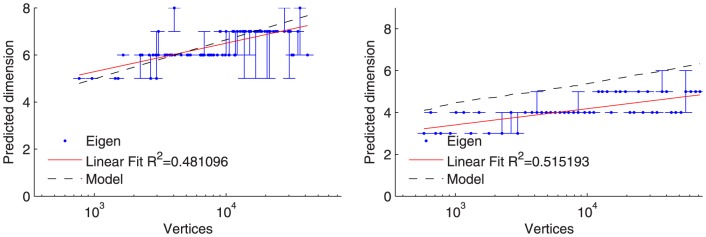
Facebook data at left, LinkedIn data at right. Each blue point (Eigen) is the dimension of the MGEO-P sample with the minimum KL-divergence between the graph and the MGEO-P sample. We also show any other other dimensions within 5% of this divergence value. The dimensions shift modestly higher for Facebook and remain almost unchanged for LinkedIn. Both still are closely correlated with the theoretical prediction based on the model based on 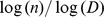 (dashed line). The linear fits to the predicted dimensions is plotted as the red linear fit line.

The results of our dimensional fitting for graphlets are shown in Figure 4 and the results of the fitting using spectral densities are in Figure 5. For both datasets and both types of statistics, the best-fit dimension scales logarithmically with the number of nodes and closely tracks a simple model prediction based on the diameter 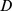 of the network (the model curve plots 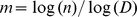). These experiments corroborate the logarithmic dimension hypothesis; although the precise fits differ as shown in Table 2.

**Table 2:** Dimension scaling for Facebook and LinkedIn.

Data	Dimension fit	Coefficients	*95% Confidence*
	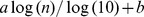	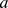	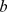	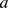	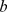
Facebook	Graphlet	2.06	−3.00	(1.851, 2.264)	(−3.821, −2.182)
	Spectral density	1.21	1.65	(0.9782, 1.446)	(0.7272, 2.578)
LinkedIn	Graphlet	0.98	1.01	(0.786, 1.178)	(0.1591, 1.87)
	Spectral density	0.77	1.1	(0.56, 0.99)	(0.23, 1.95)

The specific dimensional scaling lines fit to the data in Figures 4 and 5 illustrate the growth of the network is logarithmic in the number of nodes.

The most important feature of these results is that both methodologies show similar scaling in how the dimensionality scales with network size. There are minor differences between the precise predicted dimensions–for instance, the spectral density approach predicts slightly higher dimensions for Facebook than does the graphlet approach–but the results agree to a reasonable degree with the dimension predicted by the model: 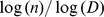. Also, the confidence bounds are small around the chosen dimension.

### Sensitivity and robustness

We investigate the sensitivity of the graphlet results in two settings. If we reduce the training set size of the SVM classifier by using a random subset of 20% of the input training data and then rerun the training and classification procedure 50 times, then we find a distribution over dimensions that we report as a box-plot, shown in Figure 4. In File S1, we further study perturbation results that argue against these results occurring due to chance. In particular, we find that these dimensions are robust to moderate changes to the network structure (Figure S2 in File S1) and we find that our methodology does not predict useful dimensions of Erdös-Rényi random graphs or random graphs with the same degree distribution (Figure 1 in File S1). We do not report a precise *p*-value as there are no widely accepted null-models for network data. We study the sensitivity of the spectral densities that look for matches that are within 105% of the true minimum divergence. This defines a dimension interval around each match that is small for all of our examples.

## Discussion

There is a growing body of evidence that argues for some type of geometric structure in social and information networks. An important study in this direction views networks as samples of geometric graphs within a hyperbolic space [25]–[27]. Recent work has further shown that hyperbolic embeddings reproduce shortest path metrics in real-world networks [28]. In both MGEO-P and hyperbolic random geometric networks, highly skewed or power-law degree distributions are imposed–either directly as in MGEO-P, or implicitly as in the hyperbolic space scaling. These results further support hidden metric structures in networks by empirically confirming a prediction about the dimension of the metric space made by one particular model. The importance of this finding is that it provides new insight into how the metric space must behave as the network grows. Previous studies assume a fixed dimension metric structure and our results indicate that a variable dimension may be more appropriate. In practice, estimating the dimensions of these networks could be useful for anomaly detection in the network and characterizing new types of network data.

**Figure 6:**
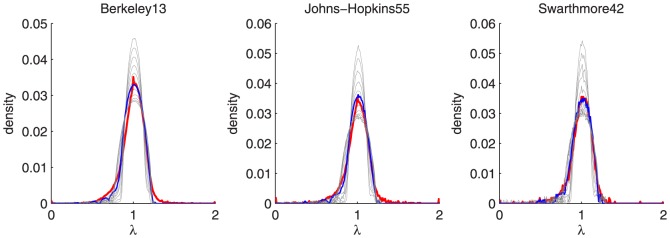
For three of the Facebook networks, we show the eigenvalue histogram in red, the eigenvalue histogram from the best fit MGEO-P network in blue, and the eigenvalue histograms for samples from the other dimensions in grey. The MGEO-P model correctly captures the peak of the distribution around 1, but fails to completely capture the tail between 1 and 2. Thus, we see meaningful difference between these profiles and hence, do not suggest that MGEO-P captures all of the properties of real-world social networks.

Note that these results do not conclusively argue that MGEO-P is a **perfectly accurate** model for social networks; there are meaningful differences between the spectral histograms from MGEO-P and real social networks, see Figure 6. There are also similar differences in the graphlet counts. Our results support a **different** hypothesis. The closest MGEO-P network to a given social network has a metric space whose dimension scales logarithmically with the number of nodes. In File S1 (Sensitivity studies section), we have determined that this property is not due to either the edge density or the degree distribution; thus, our findings appears to reflect a new intrinsic property of social networks.

This finding suggests a number of opportunities for designing social network models with metric spaces that evolve in time. We believe that such models offer the opportunity to identify new properties of social network based on emergent properties of the models. One question to address is how the metric space and connection radius change, if at all, as the network grows. Answering this question would provide insight into the value of additional users of a network. Additionally, our results suggest that many network models that assume a fixed dimension should be reevaluated.

## Materials and Methods

### Powerlaw fitting

To determine the powerlaw exponent 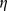, we use the Clauset-Shalizi-Newman power-law exponent estimator [29] as implemented by Tamás Nepusz [30].

### Diameters

The MGEO-P model of a network predicts that the dimension 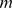 should approximate 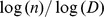, where 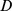 is the diamater. However, as 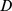 is sensitive to outliers we use the 99% effective diameter computed via an asymptotically accurate approximation scheme [31] as implemented in the SNAP library on 2011-12-31. The effective diameter of all Facebook networks ranges between 3.5 and 4.6, with a mean of 4.1. For the LinkedIn data, the effective diameter ranges between 4.3 and 5.9, with a mean of 5.4. In both networks, larger graphs have bigger effective diameters, although the differences are slight and the full data is available in the File S1, Full statistics tables.

### Graphlets

To compute graphlets, we employ the rand-esu sampling algorithm [32] as implemented in the igraph library [33]. This algorithm approximates the count of each subgraph via a stochastic search, which then depends on the probability of continuing to search. Thus, if the probability is near 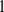 then the scores are nearly exact, but very expensive to compute, and small probabilities truncate the search early to produces fast estimates. The value we use is 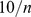. We use log-transformed output from this procedure in order to capture the dynamic range of the resulting values.

### Spectral densities

We approximate the spectral density via a 201-bin histogram of the eigenvalues of the normalized Laplacian, which all fall between 0 and 2. (The choice of 201 was based on prior experiences with the spectral histograms of networks.) To compute eigenvalues of a network, we employ the recently developed ScaLAPACK routine using the MRRR algorithm [34]–[36].

### SVM

We used a multi-class support-vector machine (SVM) based classification tool from Weka [37] to predict the relationship between the graphlets and the dimension. We considered alternatives, such as alternating decision trees and logistic regression; however, we settled on the SVM approach as it has the most flexible classification boundary to fit the highly nonlinear relationships between graphlet counts and dimensions.

### Setting MGEO-P Parameters

Consider a graph 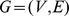 that we wish to compare to an MGEO-P sample. The MGEO-P model depends on four parameters: 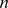, 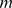, 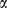, and 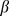. The choice of 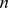 is straightforward as we use the number of nodes of the original graph. Both 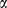 and 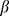 can be chosen independently of the dimension 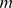. Specifically, both 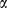 and 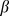 determine the average degree of the network and the exponent of the power law in the degree distribution, up to lower-order terms, as shown by property 1 and property 2. By computing just these two simple statistics of a network–the exponent of the power law and the average degree–we can invert these relationships and choose these parameters. Let 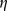 be the power-law exponent and 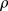 be the average degree. Then:
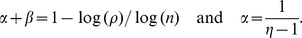



In order to derive this simple expression, we make the simplifying assumption that 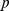 does not go to zero too quickly, for example 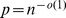, in which case: 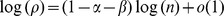 follows from the expression for the average degree of a MGEO-P network. We use the following treatment of the probability 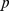 in order to maximize the clustering coefficient of the network. We first generate an MGEO-P network with 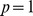. Then suppose that the original network had 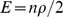 edges, we continue by randomly deleting edges until the output has exactly the same number of edges as the input network. This step can be interpreted as using the value of 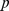 necessary to get the same edge count as the original graph. In the case where there are insufficient edges, we leave the output from the MGEO-P generator untouched. This process effectively chooses 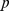 as large as possible, which gives us the largest local clustering.

## Acknowledgments

We acknowledge Jure Leskovec for allowing us to access the LinkedIn dataset.

## Supporting Information

File S1 This supporting document contains the following components of our analysis. (i) Formal proofs of the MGEO-P properties. (ii) Full statistical information about each of the Facebook and LinkedIn networks including the graphlet counts. (iii) Figure S1: Predicted dimensions of random graphs with the same degree distribution. (iv) Figure S2: The change in predicted dimension found by perturbing the graph structure. (v) A discussion of the sensitivity results about the predicted dimension.
(PDF)
