# Supplementary material for: Dimensionality of Social Networks Using Motifs and Eigenvalues
Source: PLoS One. 2014 Sep 4;9(9):e106052. doi: 10.1371/journal.pone.0106052 (PMC4154874; doi:10.1371/journal.pone.0106052)
Supplement: File S1 — This supporting document contains the following components of our analysis. (i) Formal proofs of the MGEO-P properties. (ii) Full statistical information about each of the Facebook and LinkedIn networks including the graphlet counts. (iii) Figure S1: Predicted dimensions of random graphs with the same degree distribution. (iv) Figure S2: The change in predicted dimension found by perturbing the graph structure. (v) A discussion of the sensitivity results about the predicted dimension. (PDF) [file pone.0106052.s001.pdf]

Supplemental Information for
Dimensionality of social networks using motifs and eigenvalues

Anthony Bonato,[1*] David F. Gleich,[2*] Myunghwan Kim,[3] Dieter Mitsche,[4] Paweł Prałat,[1] Yanhua Tian,[5] Stephen J. Young[6]
1 Department of Mathematics, Ryerson University
Toronto, Ontario, Canada
2 Computer Science Department, Purdue University
West Lafayette, Indiana, United States of America
3 Electrical Engineering Department, Stanford University
Stanford, California, United States of America
4 Laboratoire J.A. Dieudonné, Université de Nice Sophia-Antipolis
Nice, France
5 Department of Mathematics and Statistics, York University
Toronto, Ontario, Canada
6 Mathematics Department, University of Louisville
Louisville, Kentucky, United States of America
*To whom correspondence should be addressed.
E-mail: abonato@ryerson.ca, dgleich@purdue.edu.

Memoryless GEO-P Model

Review of GEO-P

The geometric-protean model (GEO-P) model is a model for online social networks which incorporates geometric and ranking information into an evolving network structure. More specifically, the GEO-P model, as defined by Bonato, Janssen, and Prałat [3], defines a sequence of graphs $\{G_t \colon t \geq 0\}$ on n nodes where $G_t = (V_t, E_t)$, based on four parameters: the attachment strength $\alpha \in (0, 1)$, the density parameter $\beta \in (0, 1-\alpha)$, the dimension $m \in \mathbb{N}$, and the link probability $p \in (0, 1]$. Each node $v \in V_t$ has a unique rank $r(v, t) \in [n]$ where $[n] = \{1, 2, \ldots, n\}$; we explicitly list $r(v, t)$ to emphasize that the rank may change with time. In order to stay consistent with the standard usage, the highest rank is 1 and the lowest rank is n. Additionally, each node has a geometric location in $[0, 1]^m$ under the torus metric $d(\cdot, \cdot)$. That is, for any two points $x, y \in [0, 1]^m$, $d(x, y)$ is defined to be $\min\left\{\|x - y - u\|_\infty : u \in \{-1, 0, 1\}^m\right\}$. We note that this implies that the geometric space is symmetric in any point as the metric "wraps" around. For any node v, we define its influence region at time $t \geq 0$, written $R(v, t)$, to be the ball of Euclidean volume $r(v, t)^{-\alpha} n^{-\beta}$ centered at v. Notice that, since the we are in the torus metric, this is a cube measuring $r(v, t)^{-\alpha/m} n^{-\beta/m}$ on a side.

Note. *All asymptotic results in this paper are with respect to n. We say that a statement holds* with extremely high probability*, if it holds with probability at least*

$$1 - \exp(-\omega(n) \log n)$$

for some function $\omega(n)$ with $\omega(n) \to \infty$ as $n \to \infty$. In particular, if there are a polynomial number of events, each of which holds with extremely high probability, then all of them hold with extremely high probability.

Let G_0 be any graph. In order to form G_t from G_{t-1}, first choose a node w uniformly at random from V_{t-1} and remove it. The remaining nodes are re-ranked, that is, all nodes with lower ranks than w decrease their ranks by 1. Then place a node v uniformly at random in $[0, 1]^m$, generate uniformly at random a rank for v, and re-rank the remaining nodes again. Finally, for every node u which is such that v is in the influence region of u, add the edge $\{u, v\}$ with probability p. It is clear that this process

depends only on the current state of G_t, and so forms a ergodic Markov chain with a limiting distribution π. A random instance of GEO-P is then defined to be a sample from this limiting distribution.

It is clear that the distributions of edges of G_t are determined by the relative rank histories of all the nodes at the time the other nodes entered. More specifically, if we order the nodes of G_t according to their age with node 1 being the oldest, then for any $i > j$ the probability of the edge $\{i, j\}$ being present is determined by their respective geometric locations and the rank of node j when node i arrives. Thus, in order to sample from the limiting distribution π it suffices to sample from the distributions of node histories, then randomly assign locations to the nodes, and determine if the edges are present. We note that according to the distribution π the final permutation between ages and ranks is uniformly distributed over all permutations. Since there are $n!$ permutations of nodes and at most n^2 different permutations reachable from a given state, it takes at least $\log_{n^2}(n!) = \frac{n}{2}(1 - o(1))$ iterations to reach the stationary distribution. Standard results in the mixing rate of random graphs suggest that in order to assure that a sample is close to the stationary distribution at least $\Omega\big(\log\big(\frac{n!}{n^2}\big)\big) = \Omega(n\log(n))$ iterations are required. In fact, it is easy to see that the stationary distribution is reached at the time when the last node from the initial graph G_0 is removed, which happens with probability $1 + o(1)$ after $(1 + o(1))n \log n$ steps, by the coupon collector problem.

Introducing MGEO-P

For large n this number of iterations is a significant computational roadblock, so we introduce here a variant of the GEO-P model which we call a memoryless geometric-protean graph (MGEO-P). In essence this model is the GEO-P model where the each node has forgotten its history of ranks. More specifically, a permutation σ on $[n]$ is chosen uniformly at random and $\sigma(i)$ represents the rank of the ith oldest node. Thus, for each pair $i > j$ the edge $\{i, j\}$ is potentially present if and only if the node j is in the ball of volume $\sigma(i)^{-\alpha}n^{-\beta}$ centered around node i. It is worth noting that, as shown in Bonato et al. Lemma 5.2[3], if a node in the GEO-P model receives an initial rank $R \geq \sqrt{n}\log^2 n$, then its rank is $R\left(1 + \mathcal{O}\left(\log^{-1/2}(n)\right)\right) = R(1 + o(1))$ for its entire lifetime with extremely high probability. Thus, if we imagine coupling the MGEO-P model in the natural way to GEO-P, and assuming that ranks do not change much as mentioned above, we have that for all but a vanishing fraction of the edges, the probability that a given edge is present in one model but not the other is $\mathcal{O}\big(pn^{-\alpha+2\beta/2}\log(n)^{1-4\alpha/2}\big)$. Hence, we would intuitively expect that the MGEO-P model would not differ too much from GEO-P model. In order to confirm this we prove that the parameters we are interested in do not differ by much from the proven parameters of the GEO-P model. Specifically, we look at the average degree, the degree distribution, and the diameter.

An equivalent description of the MGEO-P model

We now describe a model that is equivalent to the MGEO-P model just introduced, but that we found useful for our analysis. It has a different interpretation. The key change is that we reverse the way links are formed: when a node i arrives in the network, then all existing nodes j form links to i if i is within the influence regions of j. Intuitively, this models how links may arise in a citation network – a new paper links to those that are topically related (that is, nearby in the metric space) or highly influential. In the language we used above, this process is: fix a permutation σ on $[n]$ chosen uniformly at random and $\sigma(i)$ represents the rank of the ith oldest node. Thus, for each pair $i > j$ the edge $\{i, j\}$ is potentially present if and only if the node i is in the ball of volume $\sigma(j)^{-\alpha}n^{-\beta}$ centered around node j. The two descriptions are equivalent as we can simply reverse the order of vertex arrivals. Thus, they induce the same distribution over graphs because the order is a uniform random choice.

The average degree

In order to consider the degrees, we first need the following standard result on the tails of the hypergeometric distribution, see for instance Janson et al.[4]

Lemma 1. *Let X be the number of red balls in a set of t balls chosen at random from a set of n balls containing m red balls. Then, $\mathbb{E}[X] = \frac{tm}{n}$, and for any $\epsilon > 0$,*

$$\mathbb{P}\left(X \geq (1+\epsilon)\frac{tm}{n}\right) \leq e^{-\frac{\epsilon^2}{2+\frac{2\epsilon}{3}}\frac{tm}{n}}.$$

Further, for any $\epsilon \in (0,1)$,

$$\mathbb{P}\left(X \leq (1-\epsilon)\frac{tm}{n}\right) = e^{-\frac{\epsilon^2}{2}\frac{tm}{n}}.$$

Theorem 1. *Let $\alpha \in (0,1), \beta \in (0, 1-\alpha), n \in \mathbb{N}, m \in \mathbb{N}, p \in (0,1]$. Let v be a node of* MGEO-P(n,m,α,β,p) *with rank R and age i, then*

$$\deg(v) = \left(\frac{i-1}{n-1}\frac{p}{1-\alpha}n^{1-\alpha-\beta} + (n-i)pR^{-\alpha}n^{-\beta}\right)\left(1 + \mathcal{O}\left(\sqrt{\frac{\log^2(n)}{n^{1-\alpha-\beta}}}\right)\right),$$

with extremely high probability.

Proof. Let $\deg^+(v)$ denote the number of older neighbors of v and let $\deg^-(v)$ denote the younger neighbors of v. In order to determine $\deg^+(v)$ we consider connecting v to nodes of all ranks other than R and keeping $i-1$ of those uniformly at random. The expected degree of v before the edge deletion is

$$\sum_{r=1}^{n} pr^{-\alpha}n^{-\beta} - pR^{-\alpha}n^{-\beta} = pn^{-\beta}\int_1^n x^{-\alpha}dx + \mathcal{O}(1) = \frac{p}{1-\alpha}n^{1-\alpha-\beta} + \mathcal{O}(1).$$

Thus, $\mathbb{E}\left[\deg^+(v)\right] = \frac{i-1}{n-1}\frac{p}{1-\alpha}n^{1-\alpha-\beta} + \mathcal{O}(1)$. Furthermore, by Chernoff bounds the initial degree of v is $\frac{p}{1-\alpha}n^{1-\alpha-\beta}\left(1 + \mathcal{O}\left(\frac{\log(n)}{\sqrt{n^{1-\alpha-\beta}}}\right)\right)$ with extremely high probability, and thus, Lemma 1 gives that

$$\deg^+(v) \leq \frac{i-1}{n-1}\frac{p}{1-\alpha}n^{1-\alpha-\beta}\left(1 + \mathcal{O}\left(\sqrt{\frac{\log^2(n)n^{\alpha+\beta}}{i}}\right)\right)$$

with extremely high probability as well. Additionally, if $i \geq \log^3(n)n^{\alpha+\beta}$, then equality holds.

Since the edge probability between v and the younger nodes does not depend on the rank of the younger neighbors, $\deg^-(v)$ can be expressed as a sum of independent random variables which has expectation $(n-i)pR^{-\alpha}n^{-\beta}$. Hence, by Chernoff bounds it follows that with extremely high probability

$$\deg^-(v) \leq (n-i)pR^{-\alpha}n^{-\beta}\left(1 + \mathcal{O}\left(\sqrt{\frac{\log^2(n)R^{\alpha}n^{\beta}}{n-i}}\right)\right).$$

Now if $n - i \geq \log^3(n)R^{\alpha}n^{\beta}$, then equality holds. Combining $\deg^+(v)$ and $\deg^-(v)$ we have that with extremely high probability

$$\deg(v) \leq \frac{i-1}{n}\frac{p}{1-\alpha}n^{1-\alpha-\beta} + (n-i)pR^{-\alpha}n^{-\beta} + \mathcal{O}\left(\sqrt{\frac{\log^2(n)i}{n^{\alpha+\beta}}} + \sqrt{\frac{\log^2(n)(n-i)}{R^{\alpha}n^{\beta}}}\right).$$

In order to express the error in a multiplicative faction, we note that

$$\begin{aligned}
\frac{\log^2(n)i}{n^{\alpha+\beta}}\left(\frac{n^{\alpha+\beta}}{i-1}\right)^2 &\in \mathcal{O}\left(\frac{\log^2(n)}{n^{1-\alpha-\beta}}\right) & & i \geq \frac{n}{2}, \\
\frac{\log^2(n)i}{n^{\alpha+\beta}}\left(\frac{R^\alpha n^\beta}{n-i}\right)^2 &\in \mathcal{O}\left(\frac{\log^2(n)}{n^{1-\alpha-\beta}}\right) & & i \leq \frac{n}{2}, \\
\frac{\log^2(n)(n-i)}{R^\alpha n^\beta}\left(\frac{R^\alpha n^\beta}{n-i}\right)^2 &\in \mathcal{O}\left(\frac{\log^2(n)}{n^{1-\alpha-\beta}}\right) & & i \leq n-n\left(\frac{R}{2n}\right)^\alpha, \\
\frac{\log^2(n)(n-i)}{R^\alpha n^\beta}\left(\frac{n^\alpha n^\beta}{i-1}\right)^2 &\in \mathcal{O}\left(\frac{\log^2(n)}{n^{1-\alpha-\beta}}\right) & & i \geq n-n\left(\frac{R}{2n}\right)^\alpha.
\end{aligned}$$

Thus, for the entire range of i both of the error terms are individually dominated by one of the primary terms and hence, we have that with extremely high probability

$$\deg(v) \leq \left(\frac{i-1}{n-1}\frac{p}{1-\alpha}n^{1-\alpha-\beta} + (n-i)pR^{-\alpha}n^{-\beta}\right)\left(1+\mathcal{O}\left(\sqrt{\frac{\log^2(n)}{n^{1-\alpha-\beta}}}\right)\right),$$

and furthermore if $\log^3(n)n^{\alpha+\beta} \leq i \leq n - \log^3(n)R^\alpha n^\beta$, then equality holds.

Noting that

$$\frac{\mathbb{E}\left[\deg^+(v)\right]}{\mathbb{E}\left[\deg^-(v)\right]} = \frac{n}{n-1}\frac{i-1}{n-i}\frac{1}{1-\alpha}\left(\frac{R}{n}\right)^\alpha \in \mathcal{O}\left(\frac{\log^3(n)}{n^{1-\alpha-\beta}}\right) \subset o\left(\sqrt{\frac{\log^2(n)}{n^{1-\alpha-\beta}}}\right)$$

where $i \leq \log^3(n)n^{\alpha+\beta}$, and

$$\frac{\mathbb{E}\left[\deg^-(v)\right]}{\mathbb{E}\left[\deg^+(v)\right]} = \frac{n-1}{n}\frac{n-i}{i-1}(1-\alpha)\left(\frac{n}{R}\right)^\alpha \in \mathcal{O}\left(\frac{\log^3(n)}{n^{1-\alpha-\beta}}\right) \subset o\left(\sqrt{\frac{\log^2(n)}{n^{1-\alpha-\beta}}}\right),$$

where $n - i \geq \log^3(n)R^\alpha n^\beta$ completes the proof. □

Theorem 2. *Let* $\alpha \in (0,1), \beta \in (0, 1-\alpha), n \in \mathbb{N}, m \in \mathbb{N},$ *and* $p \in (0,1]$*, then with extremely high probability the average degree of node of* MGEO-P(n,m,α,β,p) *is*

$$d = \frac{p}{1-\alpha}n^{1-\alpha-\beta}\left(1+\mathcal{O}\left(\sqrt{\frac{\log^2(n)}{n^{1-\alpha-\beta}}}\right)\right).$$

Proof. From the proof of Theorem 1 we have that with extremely high probability for a node v with age i,

$$\deg^+(v) \leq \frac{i-1}{n-1}\frac{p}{1-\alpha}n^{1-\alpha-\beta}\left(1+\mathcal{O}\left(\sqrt{\frac{\log^2(n)}{n^{1-\alpha-\beta}}}\right)\right),$$

with equality if $i \geq \log^3(n)n^{\alpha+\beta}$. Now since every edge is counted exactly once in $\deg^+(u)$ for some node

u, the average degree is with extremely high probability

$$\begin{aligned}
\frac{2|E|}{n} &= \frac{2}{n}\sum_v \deg^+(v) \\
&\leq \frac{2}{n}\sum_{i=1}^{n} \frac{i-1}{n-1}\frac{p}{1-\alpha}n^{1-\alpha-\beta}\left(1+\mathcal{O}\left(\sqrt{\frac{\log^2(n)}{n^{1-\alpha-\beta}}}\right)\right) \\
&= \left(1+\mathcal{O}\left(\sqrt{\frac{\log^2(n)}{n^{1-\alpha-\beta}}}\right)\right)\frac{2pn^{-\alpha-\beta}}{(n-1)(1-\alpha)}\sum_{i=1}^{n}(i-1) \\
&= \left(1+\mathcal{O}\left(\sqrt{\frac{\log^2(n)}{n^{1-\alpha-\beta}}}\right)\right)\frac{2pn^{-\alpha-\beta}}{(n-1)(1-\alpha)}\binom{n}{2} \\
&= \frac{p}{1-\alpha}n^{1-\alpha-\beta}\left(1+\mathcal{O}\left(\sqrt{\frac{\log^2(n)}{n^{1-\alpha-\beta}}}\right)\right).
\end{aligned}$$

In a similar manner, we find that

$$\begin{aligned}
\frac{2|E|}{n} &\geq \left(1+\mathcal{O}\left(\sqrt{\frac{\log^2(n)}{n^{1-\alpha-\beta}}}\right)\right)\left(\frac{p}{1-\alpha}n^{1-\alpha-\beta} - \frac{2pn^{-\alpha-\beta}}{(n-1)(1-\alpha)}\binom{\log^3(n)n^{\alpha+\beta}}{2}\right) \\
&= \left(1+\mathcal{O}\left(\sqrt{\frac{\log^2(n)}{n^{1-\alpha-\beta}}}\right)\right)\left(1+\mathcal{O}\left(\frac{\log^6(n)}{n^{2-2\alpha-2\beta}}\right)\right)\frac{p}{1-\alpha}n^{1-\alpha-\beta} \\
&= \frac{p}{1-\alpha}n^{1-\alpha-\beta}\left(1+\mathcal{O}\left(\sqrt{\frac{\log^2(n)}{n^{1-\alpha-\beta}}}\right)\right),
\end{aligned}$$

completing the proof. □

The degree distribution

Let N_j be the number of nodes in MGEO-P(n,m,α,β,p) with degree precisely j and let $N_{\geq k} = \sum_{j=k}^{\infty} N_j$ be the number of nodes in degree MGEO-P(n,m,α,β,p) with degree at least k. We will show that similarly to the geometric protean graphs, $N_{\geq k} \propto k^{-\frac{1}{\alpha}}$ for a significant range of k, and thus, MGEO-P(n,m,α,β,p) exhibits a power-law degree distribution over that range with power-law exponent $1+\frac{1}{\alpha}$. Following prior work[3] we will characterize the pairs (i,R) of ages and ranks which will assure that the degree of a node is at least k and show that this value concentrates about its expectation using the following specialization of the Azuma-Hoeffding inequality.

Theorem 3 (McDiarmid's Inequality)**.** *If $X_1, X_2, \ldots, X_n$ are independent random variables and $f(x_1, x_2, \ldots, x_n)$ is a function such that for every $i \in [n]$*

$$|f(x_1,\ldots,x_i,\ldots,x_n) - f(x_1,\ldots,\hat{x}_i,\ldots,x_n)| \leq c_i,$$

then for any $\epsilon > 0$

$$\mathbb{P}(|f(X_1,X_2,\ldots,X_n) - \mathbb{E}[f(X_1,X_2,\ldots,X_n)]| > \epsilon) < 2e^{-\frac{\epsilon^2}{\sum_i c_i^2}}.$$

We will use the notation $f(n) \gg g(n)$ if $f(n)/g(n) \to \infty$ as $n \to \infty$. Similarly, $f(n) \ll g(n)$ if $g(n)/f(n) \to \infty$ as $n \to \infty$. Moreover, it will be convenient not to worry about less significant factors, so we will use $\tilde{\mathcal{O}}(f(n))$ to denote any function which is at most $f(n)\log^{O(1)} n$.

Theorem 4. *Let $\alpha \in (0,1), \beta \in (0, 1-\alpha), n \in \mathbb{N}, m \in \mathbb{N}, p \in (0,1]$, and let k and ϵ be such that $n^{1-\alpha-\beta} \ll k \ll \frac{n^{1-\frac{\alpha}{2}-\beta}}{\log^{\alpha}(n)}$, $\sqrt{\frac{\log^2(n)}{n^{1-\alpha-\beta}}}, \frac{n^{1-\alpha-\beta}}{k} \ll \epsilon$, and $\epsilon \geq c\log(n)k^{\frac{1}{\alpha}}n^{\frac{1}{2}-\frac{1-\beta}{\alpha}}$ for some $c > 0$, then with extremely high probability* MGEO-P(n, m, α, β, p) *satisfies that*

$$N_{\geq k} = (1 + \mathcal{O}(\epsilon))\frac{\alpha}{1+\alpha}p^{1/\alpha}n^{(1-\beta)/\alpha}k^{-1/\alpha}.$$

Proof. We first note that if the age rank pair (i, R) for a node v satisfies that

$$\frac{R}{n} \leq (1-\epsilon)\left(pn^{1-\alpha-\beta}\frac{n-i}{n}\frac{1}{k}\right)^{\frac{1}{\alpha}},$$

then by Theorem 1 with extremely high probability

$$\begin{aligned}
\deg(v) &= \left(\frac{i-1}{n-1}\frac{p}{1-\alpha}n^{1-\alpha-\beta} + (n-i)pR^{-\alpha}n^{-\beta}\right)\left(1 + \mathcal{O}\left(\sqrt{\frac{\log^2(n)}{n^{1-\alpha-\beta}}}\right)\right) \\
&= \left(\frac{i-1}{n-1}\frac{p}{1-\alpha}n^{1-\alpha-\beta} + \frac{n-i}{n}p\left(\frac{R}{n}\right)^{-\alpha}n^{1-\alpha-\beta}\right)\left(1 + \mathcal{O}\left(\sqrt{\frac{\log^2(n)}{n^{1-\alpha-\beta}}}\right)\right) \\
&\geq \left(\frac{i-1}{n-1}\frac{p}{1-\alpha}n^{1-\alpha-\beta} + \frac{k}{(1-\epsilon)^{\alpha}}\right)\left(1 + \mathcal{O}\left(\sqrt{\frac{\log^2(n)}{n^{1-\alpha-\beta}}}\right)\right) \\
&= \frac{k}{(1-\epsilon)^{\alpha}}(1 + o(\epsilon)) \\
&= \frac{k}{(1-\epsilon)^{\alpha}} + o(\epsilon k) \\
&= k + \alpha\epsilon k + o(\epsilon k) \\
&> k.
\end{aligned}$$

Similarly, if

$$\frac{R}{n} \geq (1+\epsilon)\left(pn^{1-\alpha-\beta}\frac{n-i}{n}\frac{1}{k}\right)^{\frac{1}{\alpha}},$$

then with extremely high probability $\deg(v) < k$.

Let X_i be the event that the node with age i has rank R satisfying

$$R \leq (1-\epsilon)n\left(pn^{1-\alpha-\beta}\frac{n-i}{n}\frac{1}{k}\right)^{\frac{1}{\alpha}},$$

and let Y_i be the event that the node with age i has rank R satisfying

$$(1-\epsilon)n\left(pn^{1-\alpha-\beta}\frac{n-i}{n}\frac{1}{k}\right)^{\frac{1}{\alpha}} \leq R \leq (1+\epsilon)n\left(pn^{1-\alpha-\beta}\frac{n-i}{n}\frac{1}{k}\right)^{\frac{1}{\alpha}}.$$

Letting $X = \sum_i X_i$ and $Y = \sum_i Y_i$ we have that $X \leq N_{\geq k} \leq X + Y$. Thus, consider

$$
\begin{aligned}
\mathbb{E}[X] &= \sum_i \mathbb{E}[X_i] \\
&= \sum_i (1-\epsilon)\left(pn^{1-\alpha-\beta}\frac{n-i}{n}\frac{1}{k}\right)^{\frac{1}{\alpha}} \\
&= (1-\epsilon)\left(\frac{pn^{-\alpha-\beta}}{k}\right)^{\frac{1}{\alpha}} \sum_i (n-i)^{\frac{1}{\alpha}} \\
&= (1-\epsilon)\left(\frac{pn^{-\alpha-\beta}}{k}\right)^{\frac{1}{\alpha}} \left(\frac{\alpha}{1+\alpha}n^{\frac{1+\alpha}{\alpha}} + \mathcal{O}(1)\right) \\
&= (1-\epsilon)\frac{\alpha}{1+\alpha}n\left(\frac{pn^{1-\alpha-\beta}}{k}\right)^{\frac{1}{\alpha}} + o\Big(\Big(\frac{\epsilon}{n}\Big)^{\frac{1}{\alpha}}\Big).
\end{aligned}
$$

We note as well that $\mathbb{E}[Y] = \frac{2\epsilon}{1-\epsilon}\mathbb{E}[X]$.

We recall that the age-rank pairs can be represented by a permutation σ chosen uniformly at random from the symmetric group, and thus, it can be generated by a sequence of transpositions $(1, a_1)(2, a_2) \cdot (n, a_n)$ where each a_i is chosen independently and uniformly at random from $\{i, i+1, \ldots, n\}$. Thus, X (and Y) may be viewed as a function of independent random variables and so Theorem 3 applies. Furthermore, the change of any particular variable impacts the value of X by at most 2. Hence, we note that,

$$
\frac{1}{n}\epsilon^2\mathbb{E}[X]^2 \geq \epsilon^2(1-\epsilon)^2\frac{\alpha^2}{(1+\alpha)^2}n\left(\frac{pn^{1-\alpha-\beta}}{k}\right)^{\frac{2}{\alpha}} \in \Omega\big(\log^2(n)\big),
$$

and thus, with extremely high probability $|X - \mathbb{E}[X]| \leq \epsilon\mathbb{E}[X]$ and $|Y - \mathbb{E}[Y]| \leq \epsilon\mathbb{E}[X]$. Hence, we have that

$$
\mathbb{E}[X] - \epsilon\mathbb{E}[X] \leq N_{\geq k} \leq \mathbb{E}[X] + 3\epsilon\mathbb{E}[X],
$$

with extremely high probability and the desired result follows. □

We note that by choosing $\epsilon = \log^{-1/3}(n)$ we can easily obtain the same type of degree distribution result for MGEO-P that exists for the original GEO-P[3].

Theorem 5. *Let $\alpha \in (0,1), \beta \in (0, 1-\alpha), n \in \mathbb{N}, m \in \mathbb{N}, p \in (0,1]$, and*

$$
n^{1-\alpha-\beta}\log^{1/2}(n) \leq k \leq n^{1-\alpha/2-\beta}\log^{-2\alpha-1}(n),
$$

then with extremely high probability MGEO-P(n, m, α, β, p) *satisfies*

$$
N_{\geq k} = \Big(1 + \mathcal{O}\Big(\log^{-1/3}(n)\Big)\Big)\frac{\alpha}{1+\alpha}p^{1/\alpha}n^{(1-\beta)/\alpha}k^{-\frac{1}{\alpha}},
$$

where $N_{\geq k}$ is the number of nodes of degree at least k.

The diameter

Theorem 6. *Let $\alpha \in (0,1), \beta \in (0, 1-\alpha), n \in \mathbb{N}, m \in \mathbb{N}, p \in (0,1]$. The diameter of* MGEO-P(n, m, α, β, p) *is $n^{\Theta\left(\frac{1}{m}\right)}$ with extremely high probability.*

Proof. We first show that the diameter is $\tilde{\mathcal{O}}\left(n^{\frac{\beta}{(1-\alpha)m}}\right) \in n^{\mathcal{O}\left(\frac{1}{m}\right)}$. To this end, let

$$t = 10\left\lfloor\left(n^{\frac{\beta}{(1-\alpha)}}\ln^{\frac{2\alpha}{1-\alpha}}(n)\right)^{1/m}\right\rfloor$$

and divide $[0,1)^m$ into t^m uniform subcubes with side-lengths $\frac{1}{t}$ in the natural way. Now, as $\frac{\beta}{(1-\alpha)} < 1$, by Chernoff bounds there are $\tilde{\Theta}\left(n^{1-\frac{\beta}{(1-\alpha)}}\right)$ nodes in each of the subcubes with extremely high probability. Thus, in order to show diameter $\tilde{\mathcal{O}}\left(n^{\frac{\beta}{(1-\alpha)m}}\right)$ it suffices to show that for any two nodes u and v at ℓ_∞-distance at most $2/t$ the graph distance between the two nodes is at most some fixed constant.

Now consider an arbitrary node v. By Chernoff bounds, with extremely high probability there are $\Omega\left(n^{1-\alpha-\beta}\right)$ nodes at ℓ_∞ distance at most $\frac{1}{2}n^{-\frac{\alpha+\beta}{m}}$ from v and age rank at least $\frac{n}{2}$ (that is, young nodes). As the radius of influence of every node is at least $\frac{1}{2}n^{-\frac{\alpha+\beta}{m}}$, this implies that with extremely high probability every node has $\Omega\left(n^{1-\alpha-\beta}\right)$ neighbors at ℓ_∞-distance at most $\frac{1}{2}n^{-\frac{\alpha+\beta}{m}}$ with age rank at least $\frac{n}{2}$.

In a similar manner, by combining Lemma 1 and Chernoff bounds, with extremely high probability every node v with age rank at least $\frac{n}{2}$ has

$$\Omega\Big((1/t)^m n^{\beta/(1-\alpha)}\ln^{2/(1-\alpha)} n\Big) = \Omega\Big(\ln^{-2\alpha/(1-\alpha)+2/(1-\alpha)} n\Big) = \Omega\big(\ln^2 n\big)$$

neighbors at ℓ_∞-distance at most $\frac{1}{2t}$, with rank at most $n^{\frac{\beta}{1-\alpha}}\ln^{2/(1-\alpha)} n$, and age rank at most $n/2$ (that is, old nodes). Note that each node with rank at most $n^{\frac{\beta}{1-\alpha}}\ln^{2/(1-\alpha)} n$ has radius of influence at least

$$\frac{1}{2}\left(n^{\frac{-\alpha\beta}{1-\alpha}-\beta}\ln^{-2\alpha/(1-\alpha)} n\right)^{1/m} = (1+o(1))\frac{5}{t}.$$

Combining these two observations we have that, with extremely high probability, every node v is within graph-distance two and ℓ_∞-distance $n^{-\frac{\alpha+\beta}{m}} + \frac{1}{2t}$ of a set of $\Theta\big(\ln^2(n)\big)$ nodes, X_v, with rank at most $n^{\frac{\beta}{1-\alpha}}\ln^{2/(1-\alpha)} n$. Thus, if u and v are at ℓ_∞-distance at most $\frac{2}{t}$, the distance between elements of X_u and X_v is at most $\frac{2}{t} + 2n^{-\frac{\alpha+\beta}{m}} + \frac{1}{t} \le \frac{4}{t}$. On the other hand, as we already mentioned, the radius of influence of each node in X_u or X_v is at least $4/t$. Thus, with extremely high probability, some member of X_u and X_v are adjacent and hence u and v are within graph distance 5, completing the proof of the upper bound.

For the lower bound, let us take some node v and consider distances to other nodes. With probability $1-2^{-m}$ some other node is at ℓ_∞-distance at least $1/4$. Hence, by Chernoff bounds, with extremely high probability there exist two nodes at ℓ_∞-distance at least $\frac{1}{4}$. As the diameter of every influence region is at most $n^{-\frac{\beta}{m}}$, this gives that the diameter of the graph is $\Omega\left(n^{\frac{\beta}{m}}\right) \in n^{\Omega(1/m)}$. □

Experimental design

Given a graph $G = (V, E)$, we employ the following methods to determine the dimension m of the MGEO-P models:

Experiment 1
1. Set n to the number of nodes. Determine values of α and β independently of m (see the appendix of the original paper).
2. Simulate 50 samples of an MGEO-P network with m varying between 1 and 12.

3. Compute the graphlet counts for each sample of MGEO-P and train a SVM classifier to predict the dimension of the network given the samples.
4. Compute the graphlet counts for the graph G and use the output from the classifier as the dimension m of the network.

Experiment 2
1 & 2. As in experiment 1.
3. Compute the spectral density for one sample of MGEO-P for each m between 1 and 12 (only one MGEO-P sample is used to get the density).[1]
4. Compute the spectral density of the graph G and find the value of m that minimizes the KL-divergence between the density from the graph and the MGEO-P samples.

The first approach employs a complex statistical technique—the support vector machine classifier—to determine nonlinear predictive correlations among the graphlet counts and the dimension. This sophistication renders the method opaque and difficult to interpret the precise similarity mechanism. The second approach is simple and still illustrates the dimensional scaling, although the precise dimensions differ, which indicates that it is matching the network in a different way.

Estimating dimensions using graphlets and support vector machines

The relationship between the dimension of a graph and its graphlets is highly nonlinear and so we used a multi-class support-vector machine (SVM) based classification tool from WEKA to predict this relationship. In this case, each dimension is a class, but as an SVM can only make a binary decision we train the SVM using a dimension-vs-dimension classification. That is, we build a classifer to predict dimension 5-vs-dimension 3, dimension 5-vs-dimension 4, etc. so there are 66 = “12-choose-2” SVMs trained. The dimension picked most often among these classifiers is the predicted class; this is the standard behavior of the sequential minimal optimization classifier (SMO) used in Weka. The dimension of a real-world network is then predicted by running this classifier on the graphlet counts of the networks. An alternative methodology (which has had some previous success) would be to to train the classifier using alternating decision trees; however this training methodology significantly restricts the behavior of the classifier and produces inconsistent results.

Comparing spectral densities

Given the eigenvalues of the normalized Laplacian, we compute a spectral density by taking a 201-bin histogram of these eigenvalues. We then use the KL-divergence between these histograms as used in Banerjee and Jost (2009) as a measure of similarity. If P^A and P^B are the histograms of networks A and B normalized to probabilities, then for our 201-bin histograms we have that:

$$KL(A,B) = \sum_{i=1}^{201} \log(P_i^A / P_i^B) P_i^A.$$

We select the single best dimension based on the value of m that minimizes the KL divergence $KL(S, G_m)$ where S is the sample of either Facebook or LinkedIn and G_m is a sample of a MGEO-P network with dimension m. We add 1 to all of the eigenvalue counts in the histogram as a form of smoothing for the probabilities. We define a dimension interval by looking at the maximum interval such that the extreme points are within 105% of the true minimum.

[1] We only use one sample of the MGEO-P network to estimate the eigenvalue distribution as these computations are time-consuming and our preliminary studies showed that the spectral density had only small variations between repeated samples.

Sensitivity studies

In the following sections, we study how the predicted dimension changes due to large scale structural changes in the graph. We focus our efforts on studying the Facebook samples as the LinkedIn samples are highly correlated due to the temporal nature of their construction. Our results show that

1. Erdős Rényi random graphs have no apparent dimension.
2. The graphlet fitting methodology is influenced by the degree distribution in a way that generates high variance in the predicted dimension but where a logarithmic trend may still exist. This effect is not present in the spectral histograms.
3. The graphlet fitting methodology is robust to changing 10% of the edges of the network via a random percolation process.

Dimensions of Erdős Rényi random graphs

In our first experiment to verify the relevance of our dimensionality fits, we attempt to fit the dimension of an Erdős Rényi random graph with the same number of expected edges. That is, for each of the samples of the Facebook network, we run the SVM dimension classifier we constructed on the graphlet counts of 50 separate Erdős Rényi random graph samples where the probability is designed to yield the number of edges of the original network in expectation. In all but 3 of the 5000 examples (50 samples for each of the 100 graphs), the predicted dimension is the maximum 12. When the dimension was not the maximum in those three cases, it was 11. When we tried this with dimensions up to 10, then the Erdős Rényi random graphs fit to the dimension 10, thus, we expect these graphs to be predicted at the highest dimension of the training set. We see this as evidence that our graphlet methodology is sensitive to clearly erroneous graphs.

Dimensions of random graphs with the same degree distribution

In our second experiment to verify the relevance of our dimension fits, we attempt to fit the dimension of a graph with the same degree distribution as one of the Facebook networks but with edges randomly drawn. To generate these graphs, we use the Bayati-Saberi-Kim procedure[1] as implemented in the bisquik library[2]. This method terminated for 92 of the 100 graphs. (The process did not terminate in the other 8 cases, which is a limitation of this particular sampling scheme.) The dimensional fits for these 92 resampled networks are shown in Figure S1. The eigenvalue fits show no logarithmic scaling in the dimension whereas the graphlet fits do. However, the variance in the predicted dimensions based on graphlets is substantially higher for these random samples compared to the original networks (see Figure 4 in the main text). The evidence from graphlets alone, is then, possibly biased due to the degree distribution. However, the results from the spectral histograms, the graphlets, and the prediction dimension from the model itself encourage us to be more optimistic.

Dimension variance with random percolation

In our final experiment, we study random percolation of the predicted dimension of the Facebook networks. In a random percolation process, we randomly sample an edge from the network, delete it, replace it with an edge between two randomly drawn nodes, and continue until we have done this procedure k times. We study how the predicted dimension varies as we change 1%, 5%, 10%, 15%, 20%, 25%, 30%, 35%, 40%, 45%, 50% of the total edges of a network. For each of the 100 Facebook networks, and each

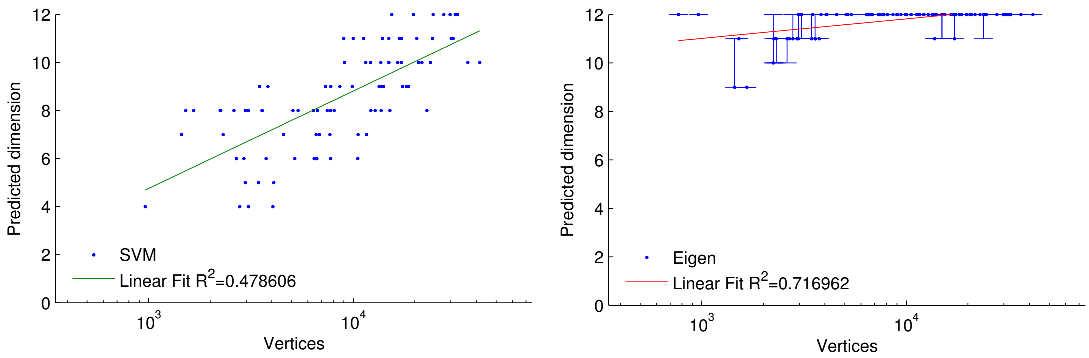

Figure S1: Predicted dimensions of random graphs with the same degree distribution. At left, the predicted dimension using our SVM-Graphlet methodology and at right, the prediction dimension using our spectral histogram methodology

percentage of total edges, we repeat the percolation process 10 times. This generates 110 total networks for each Facebook network. Figure S2 shows a box-plot of how the predicted dimension varies for each perturbation level over all 1100 total graphs. This plot suggests that the dimension is unchanged until more than 15% of the edges have been percolated. This figure further illustrates that the predicted dimension is a stable quantity for a network that is not overly sensitive to small perturbations.

Online codes

Our experimental codes and data for the graphlet and spectral densities are available from

`https://www.cs.purdue.edu/homes/dgleich/codes/geop-dim/`

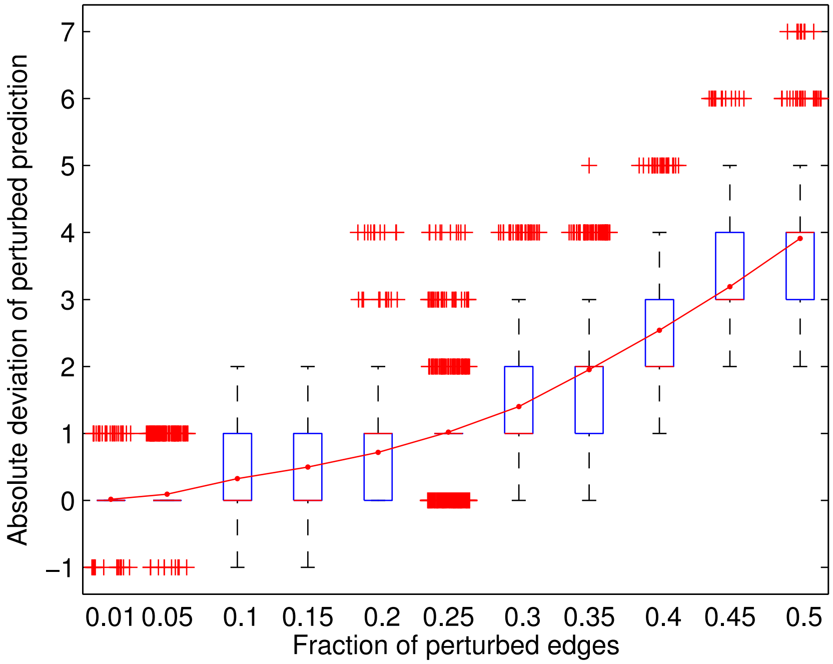

Figure S2: The change in the predicted dimension based on the graphlets methodology as we randomly percolate small or large fractions of the total edges in the network. Each box-plot represents the results over all 100 Facebook networks. The label 0.05 corresponds to randomly altering 5% of the total edges in a network. The line tracks the mean over all the samples.

Full statistics of Facebook data

Table of properties of the Facebook networks.

Name	Nodes	Edges	PL Exp	Eff. Diam	α	β	Dim_G	Dim_E
Caltech36	769	16656	7.00	3.81	0.17	0.27	4	5
Reed98	962	18812	4.38	3.88	0.30	0.17	3	5
Haverford76	1446	59589	7.00	3.63	0.17	0.23	4	5
Simmons81	1518	32988	4.74	3.92	0.27	0.22	4	5
Swarthmore42	1659	61050	5.60	3.77	0.22	0.20	3	6
Amherst41	2235	90954	5.64	3.81	0.22	0.21	3	6
Bowdoin47	2252	84387	5.80	3.81	0.21	0.23	4	6
Hamilton46	2314	96394	4.63	3.79	0.28	0.15	4	6
Trinity100	2613	111996	5.83	3.84	0.21	0.23	4	6
USFCA72	2682	65252	4.13	3.97	0.32	0.19	4	5
Williams40	2790	112986	5.17	3.82	0.24	0.21	4	6
Oberlin44	2920	89912	4.83	3.96	0.26	0.22	4	6
Smith60	2970	97133	5.78	3.84	0.21	0.27	4	6
Wellesley22	2970	94899	4.64	3.92	0.27	0.21	4	5
Vassar85	3068	119161	5.93	3.82	0.20	0.26	4	6
Middlebury45	3075	124610	6.59	3.92	0.18	0.27	4	7
Pepperdine86	3445	152007	5.27	3.88	0.23	0.22	4	6
Colgate88	3482	155043	6.07	3.81	0.20	0.25	4	6
Santa74	3578	151747	5.74	3.90	0.21	0.25	4	6
Wesleyan43	3593	138035	4.72	3.92	0.27	0.20	4	6
Mich67	3748	81903	5.03	4.26	0.25	0.29	5	6
Bucknell39	3826	158864	5.70	3.85	0.21	0.25	4	6
Brandeis99	3898	137567	4.98	3.85	0.25	0.23	5	6
Howard90	4047	204850	6.48	3.81	0.18	0.26	4	8
Rice31	4087	184828	6.30	3.82	0.19	0.27	4	6
Rochester38	4563	161404	5.34	3.96	0.23	0.27	5	6
Lehigh96	5075	198347	6.40	3.90	0.19	0.30	4	6
Johns-Hopkins55	5180	186586	5.57	4.07	0.22	0.28	5	6
Wake73	5372	279191	5.71	3.86	0.21	0.25	4	6
American75	6386	217662	4.85	4.07	0.26	0.26	5	6
MIT8	6440	251252	5.24	4.02	0.24	0.27	5	6
William77	6472	266378	5.19	3.90	0.24	0.26	5	6
UChicago30	6591	208103	4.80	4.27	0.26	0.27	5	6
Princeton12	6596	293320	5.35	4.00	0.23	0.26	5	6
Carnegie49	6637	249967	4.98	4.04	0.25	0.26	5	6
Tufts18	6682	249728	5.48	4.22	0.22	0.29	5	6
UC64	6833	155332	5.71	4.58	0.21	0.36	5	6
Vermont70	7324	191221	4.72	4.24	0.27	0.29	5	6
Emory27	7460	330014	6.18	4.02	0.19	0.30	5	6
Dartmouth6	7694	304076	5.45	4.11	0.22	0.29	5	6
Tulane29	7752	283918	6.64	4.10	0.18	0.34	5	6
WashU32	7755	367541	5.23	3.94	0.24	0.26	5	6
Villanova62	7772	314989	5.58	4.09	0.22	0.29	5	6
Vanderbilt48	8069	427832	5.61	3.91	0.22	0.26	5	6
Yale4	8578	405450	5.79	4.02	0.21	0.29	5	6
Brown11	8600	384526	4.85	4.01	0.26	0.24	5	6
UCSC68	8991	224584	5.11	4.56	0.24	0.33	5	7
Maine59	9069	243247	5.25	4.29	0.24	0.33	6	7
Georgetown15	9414	425638	4.91	4.13	0.26	0.25	5	6
Duke14	9895	506442	5.52	4.02	0.22	0.28	5	6
Bingham82	10004	362894	5.96	4.08	0.20	0.33	5	7
Mississippi66	10521	610911	5.46	3.90	0.22	0.26	5	6
Northwestern25	10567	488337	5.65	3.98	0.22	0.30	5	6

Name	Nodes	Edges	PL Exp	Eff. Diam	α	β	Dim_G	Dim_E
Cal65	11247	351358	7.00	4.33	0.17	0.39	5	7
BC17	11509	486967	5.41	4.13	0.23	0.30	5	7
Stanford3	11621	568330	5.76	4.31	0.21	0.30	5	6
Columbia2	11770	444333	4.29	4.37	0.30	0.24	6	6
Notre-Dame57	12155	541339	4.83	4.01	0.26	0.26	6	7
GWU54	12193	469528	4.76	4.14	0.27	0.27	6	7
Baylor93	12803	679817	5.81	3.90	0.21	0.30	5	7
USF51	13377	321214	4.93	4.65	0.25	0.34	5	7
Syracuse56	13653	543982	5.93	4.08	0.20	0.34	5	7
Temple83	13686	360795	4.35	4.52	0.30	0.29	6	7
UC61	13746	442174	5.33	4.47	0.23	0.33	5	7
Northeastern19	13882	381934	4.39	4.54	0.29	0.28	6	7
JMU79	14070	485564	5.20	3.98	0.24	0.32	6	7
UPenn7	14916	686501	5.89	4.15	0.20	0.32	5	7
UCSB37	14935	482224	5.54	4.49	0.22	0.35	5	7
UCF52	14940	428989	5.51	4.28	0.22	0.36	6	7
UCSD34	14948	443221	5.01	4.46	0.25	0.33	6	7
Harvard1	15126	824617	5.69	4.41	0.21	0.30	5	7
MU78	15436	649449	6.21	4.08	0.19	0.35	6	7
UMass92	16516	519385	4.54	4.15	0.28	0.29	6	7
UC33	16808	522147	4.79	4.46	0.26	0.31	6	7
Tennessee95	16979	770659	4.83	4.05	0.26	0.28	6	7
UVA16	17196	789321	5.45	4.00	0.22	0.31	6	7
UConn91	17212	604870	5.15	4.09	0.24	0.32	6	7
Oklahoma97	17425	892528	5.26	3.97	0.23	0.29	5	7
USC35	17444	801853	6.31	4.09	0.19	0.35	6	7
UNC28	18163	766800	4.45	3.99	0.29	0.26	7	7
Auburn71	18448	973918	4.99	3.92	0.25	0.28	6	7
Cornell5	18660	790777	5.10	4.34	0.24	0.31	6	7
BU10	19700	637528	5.32	4.49	0.23	0.35	6	7
UCLA26	20467	747613	5.67	4.51	0.21	0.35	6	7
Maryland58	20871	744862	5.41	4.19	0.23	0.34	6	7
Virginia63	21325	698178	4.54	4.14	0.28	0.30	6	7
NYU9	21679	715715	4.15	4.42	0.32	0.26	6	7
Berkeley13	22937	852444	4.32	4.32	0.30	0.27	6	7
Wisconsin87	23842	835952	4.74	4.30	0.27	0.31	6	7
UGA50	24389	1174057	5.77	3.99	0.21	0.34	6	7
Rutgers89	24580	784602	5.33	4.60	0.23	0.36	6	7
FSU53	27737	1034802	5.79	4.45	0.21	0.37	6	7
Indiana69	29747	1305765	5.40	4.22	0.23	0.34	6	7
Michigan23	30147	1176516	5.12	4.53	0.24	0.33	6	7
UIllinois20	30809	1264428	5.56	4.35	0.22	0.35	6	7
Texas80	31560	1219650	5.65	4.44	0.22	0.37	6	6
MSU24	32375	1118774	5.11	4.38	0.24	0.35	6	6
UF21	35123	1465660	4.92	4.17	0.26	0.32	6	7
Texas84	36371	1590655	4.79	4.08	0.26	0.31	6	8
Penn94	41554	1362229	4.16	4.56	0.32	0.29	7	6

Table of log-graphlets counts of the Facebook networks

Name	$\tilde{G}_1$	$\tilde{G}_2$	$\tilde{G}_3$	$\tilde{G}_4$	$\tilde{G}_5$	$\tilde{G}_6$	$\tilde{G}_7$	$\tilde{G}_8$
Caltech36	13.718	11.731	17.388	17.766	16.802	13.808	14.806	13.148
Reed98	13.902	11.560	17.884	18.200	17.004	14.272	14.729	12.498
Haverford76	15.514	13.307	19.375	20.127	18.868	16.273	16.775	14.728

Name	$\tilde{G}_1$	$\tilde{G}_2$	$\tilde{G}_3$	$\tilde{G}_4$	$\tilde{G}_5$	$\tilde{G}_6$	$\tilde{G}_7$	$\tilde{G}_8$
Simmons81	14.421	12.015	18.476	18.950	17.604	14.759	15.341	13.296
Swarthmore42	15.558	13.229	19.853	20.360	19.099	16.372	16.884	14.733
Amherst41	15.964	13.672	20.383	20.974	19.725	16.908	17.565	15.582
Bowdoin47	15.868	13.491	20.066	20.694	19.302	16.519	17.053	15.091
Hamilton46	16.075	13.724	20.384	20.950	19.552	16.808	17.239	15.129
Trinity100	16.201	13.887	20.534	21.243	19.833	17.065	17.596	15.634
USFCA72	15.404	12.859	19.837	20.414	18.884	16.018	16.458	14.442
Williams40	16.270	13.833	20.883	21.393	20.029	17.129	17.696	15.621
Oberlin44	15.860	13.215	20.484	20.975	19.351	16.442	16.813	14.691
Smith60	15.860	13.325	20.315	20.981	19.411	16.281	16.912	15.261
Wellesley22	15.993	13.350	20.594	21.118	19.533	16.633	16.993	14.840
Vassar85	16.294	13.655	20.725	21.409	19.774	17.012	17.262	15.029
Middlebury45	16.338	13.914	20.785	21.454	19.995	17.117	17.672	15.709
Pepperdine86	16.763	14.319	21.290	21.864	20.403	17.543	18.043	16.114
Colgate88	16.570	14.120	20.940	21.675	20.159	17.317	17.827	15.916
Santa74	16.642	14.183	21.265	21.854	20.362	17.468	17.996	16.035
Wesleyan43	16.523	14.002	20.967	21.619	20.035	17.142	17.618	15.653
Mich67	15.523	12.990	20.435	20.894	19.409	16.214	16.944	15.171
Bucknell39	16.604	14.135	21.011	21.762	20.236	17.303	17.879	16.079
Brandeis99	16.584	13.875	23.371	21.924	21.023	17.326	18.008	15.722
Howard90	17.223	14.542	22.062	22.623	20.957	18.341	18.425	16.117
Rice31	16.950	14.489	21.533	22.186	20.657	17.556	18.237	16.396
Rochester38	16.592	14.103	21.867	21.935	20.429	17.335	17.966	16.241
Lehigh96	16.849	14.300	21.824	22.269	20.725	17.709	18.273	16.449
Johns-Hopkins55	16.825	14.287	22.032	22.336	20.841	17.745	18.391	16.594
Wake73	17.496	15.025	22.285	22.886	21.320	18.248	18.895	17.249
American75	16.981	14.238	21.921	22.521	20.751	17.591	18.095	16.398
MIT8	17.263	14.629	22.254	22.856	21.200	18.098	18.656	16.864
William77	17.297	14.587	23.076	23.038	21.438	18.222	18.679	16.888
UChicago30	16.832	14.068	22.312	22.533	20.798	17.599	18.102	16.351
Princeton12	17.450	14.720	22.371	23.056	21.296	18.367	18.714	16.637
Carnegie49	17.195	14.619	22.400	22.890	21.264	18.152	18.713	16.857
Tufts18	17.178	14.458	22.155	22.758	20.978	17.908	18.353	16.427
UC64	16.320	13.797	21.212	21.792	20.205	16.810	17.797	16.375
Vermont70	16.550	13.750	21.667	22.193	20.403	17.205	17.741	15.907
Emory27	17.508	14.937	22.487	23.133	21.521	18.507	19.140	17.365
Dartmouth6	17.437	14.626	22.655	23.152	21.351	18.337	18.757	16.699
Tulane29	17.287	14.752	22.379	22.950	21.338	18.099	18.891	17.347
WashU32	17.751	15.070	24.290	23.744	22.343	18.961	19.492	17.472
Villanova62	17.474	14.767	22.473	23.104	21.376	18.316	18.806	16.948
Vanderbilt48	18.003	15.401	23.641	23.678	22.035	18.877	19.431	17.748
Yale4	17.867	15.043	23.511	23.710	21.951	18.763	19.200	17.250
Brown11	17.739	14.846	23.016	23.580	21.716	18.701	19.047	17.014
UCSC68	16.615	13.980	21.546	22.232	20.499	17.054	17.956	16.316
Maine59	16.843	13.954	22.256	22.519	20.624	17.278	17.875	16.161
Georgetown15	17.875	15.029	23.093	23.689	21.824	18.778	19.132	17.152
Duke14	18.194	15.468	23.419	24.008	22.262	19.163	19.713	17.913
Bingham82	17.416	14.647	22.543	23.260	21.441	18.197	18.915	17.258
Mississippi66	18.525	15.924	24.072	24.494	22.847	19.665	20.335	18.567
Northwestern25	18.054	15.302	24.403	24.064	22.399	19.015	19.570	17.727
Cal65	17.326	14.567	22.509	23.209	21.433	17.982	18.757	17.390
BC17	17.905	14.992	23.342	23.902	22.019	18.969	19.366	17.386
Stanford3	18.314	15.527	23.744	24.356	22.520	19.306	19.783	18.121
Columbia2	18.027	15.042	24.979	24.149	22.584	19.149	19.705	17.484
Notre-Dame57	18.120	15.088	23.689	24.111	22.105	18.935	19.317	17.287
GWU54	17.944	15.032	24.613	24.060	22.284	18.838	19.363	17.599
Baylor93	18.529	15.734	24.456	24.655	22.822	19.610	20.119	18.376
USF51	17.259	14.443	22.687	23.190	21.281	18.062	18.569	16.929

Name	$\tilde{G}_1$	$\tilde{G}_2$	$\tilde{G}_3$	$\tilde{G}_4$	$\tilde{G}_5$	$\tilde{G}_6$	$\tilde{G}_7$	$\tilde{G}_8$
Syracuse56	17.948	15.270	23.999	24.101	22.329	18.987	19.663	18.297
Temple83	17.470	14.446	23.621	23.581	21.669	18.532	18.860	16.869
UC61	17.692	15.061	23.205	23.766	22.100	18.442	19.376	17.989
Northeastern19	17.324	14.343	22.931	23.363	21.360	18.038	18.618	16.892
JMU79	17.826	14.817	25.055	23.911	22.157	18.468	19.027	17.504
UPenn7	18.403	15.520	23.941	24.550	22.617	19.369	19.867	18.070
UCSB37	17.768	14.983	23.076	23.754	21.915	18.352	19.210	17.784
UCF52	17.936	15.121	25.126	23.785	22.209	18.435	19.460	18.270
UCSD34	17.596	14.777	23.748	23.679	21.847	18.195	19.061	17.590
Harvard1	18.857	15.913	24.389	25.025	23.095	20.079	20.333	18.188
MU78	18.186	15.370	23.543	24.261	22.387	18.844	19.641	18.263
UMass92	17.800	14.756	24.903	23.986	22.100	18.601	19.198	17.446
UC33	17.831	14.971	23.489	23.945	22.109	18.467	19.340	17.816
Tennessee95	18.822	15.821	25.122	24.846	22.964	19.566	20.153	18.553
UVA16	18.681	15.691	24.568	24.786	22.854	19.422	20.060	18.381
UConn91	18.012	15.073	23.583	24.141	22.113	18.638	19.347	17.725
Oklahoma97	18.956	16.166	24.691	25.130	23.322	19.898	20.686	19.128
USC35	18.681	15.760	24.776	24.863	22.972	19.538	20.228	18.667
UNC28	18.724	15.544	24.894	24.532	22.325	18.877	19.196	17.272
Auburn71	19.105	16.140	25.604	25.360	23.539	19.947	20.678	19.123
Cornell5	18.598	15.614	25.378	24.834	22.939	19.449	20.112	18.464
BU10	18.035	14.925	23.718	24.231	22.137	18.678	19.259	17.708
UCLA26	18.331	15.435	23.907	24.597	22.657	19.068	19.866	18.416
Maryland58	18.376	15.374	25.593	24.728	22.783	19.165	19.773	18.264
Virginia63	18.448	15.322	26.598	24.835	23.067	19.158	19.911	18.418
NYU9	18.307	15.117	24.927	24.656	22.562	19.064	19.574	17.772
Berkeley13	18.637	15.458	26.726	25.366	23.590	19.668	20.406	18.426
Wisconsin87	18.472	15.374	25.177	24.862	22.817	19.153	19.957	18.362
UGA50	19.010	16.124	24.970	25.418	23.515	19.869	20.751	19.238
Rutgers89	18.244	15.303	24.316	24.585	22.615	18.987	19.843	18.299
FSU53	18.729	15.938	24.706	25.089	23.220	19.551	20.543	19.192
Indiana69	18.989	16.028	24.958	25.537	23.496	19.971	20.731	19.257
Michigan23	18.861	15.914	25.187	25.433	23.418	19.984	20.730	19.046
UIllinois20	18.957	16.040	25.801	25.446	23.498	19.865	20.728	19.296
Texas80	18.905	16.092	24.841	25.387	23.520	19.807	20.845	19.340
MSU24	18.829	15.676	25.554	25.234	23.194	19.711	20.328	18.660
UF21	19.324	16.295	27.899	26.069	24.224	20.261	21.154	19.737
Texas84	19.574	16.232	26.785	26.199	24.047	20.229	20.911	19.327
Penn94	19.106	15.797	26.853	25.886	23.738	19.985	20.750	18.978

Full statistics of LinkedIn data

Table of properties of the LinkedIn networks. We only compute eigenvalue dimensional fits up to $72,000$ nodes.

Nodes	Edges	PL Exp	Eff. Diam	α	β	Dim_G	Dim_E
578	3140	3.01	4.70	0.50	0.14	4	3
650	3596	3.02	4.75	0.50	0.13	4	4
709	4032	2.93	4.68	0.52	0.12	3	3
774	4448	2.77	4.68	0.56	0.07	3	3
916	5300	2.74	4.71	0.57	0.07	3	3
1012	5983	2.82	4.69	0.55	0.10	4	4
1158	6955	2.77	4.77	0.56	0.08	4	4
1312	7957	2.75	4.79	0.57	0.08	4	3
1512	9287	2.80	4.83	0.56	0.11	4	4
1800	11366	2.73	4.93	0.58	0.09	4	3
2259	14643	2.66	5.07	0.60	0.08	4	3
2643	17795	2.74	5.06	0.57	0.10	4	4
2972	20465	2.61	5.10	0.62	0.06	4	3
3389	23432	2.76	5.20	0.57	0.12	4	4
3817	26656	2.82	5.28	0.55	0.14	5	4
4158	29086	2.81	5.23	0.55	0.14	4	4
4508	31557	2.80	5.30	0.56	0.13	4	4
4898	34273	2.81	5.37	0.55	0.15	5	4
5288	37173	2.85	5.30	0.54	0.15	5	4
5728	40550	2.86	5.36	0.54	0.16	5	4
6216	44091	2.85	5.40	0.54	0.16	4	4
6719	47813	2.81	5.47	0.55	0.15	5	4
7230	51600	2.84	5.50	0.54	0.16	5	4
7892	56238	2.83	5.47	0.55	0.16	5	4
8551	60985	2.87	5.56	0.53	0.17	5	4
9572	68113	2.88	5.54	0.53	0.18	5	4
10444	74215	2.90	5.55	0.53	0.19	5	4
11222	79805	2.91	5.58	0.52	0.19	5	4
12210	87129	2.93	5.57	0.52	0.20	5	5
13043	93158	2.95	5.59	0.51	0.21	5	5
14173	101279	3.22	5.59	0.45	0.27	6	5
15246	109186	2.94	5.59	0.52	0.21	5	4
16499	118284	3.22	5.62	0.45	0.28	6	5
17816	127765	3.23	5.65	0.45	0.28	5	5
19522	140191	3.28	5.67	0.44	0.29	6	5
21125	151651	2.79	5.68	0.56	0.18	6	4
22665	163457	2.79	5.66	0.56	0.18	5	4
24530	177418	3.38	5.69	0.42	0.32	7	5
26535	191947	2.87	5.72	0.53	0.21	5	4
28760	207848	3.78	5.72	0.36	0.38	7	5
31176	225897	2.84	5.77	0.54	0.20	6	4
33834	246160	2.84	5.79	0.54	0.20	5	4
37010	270718	3.39	5.78	0.42	0.33	6	5
40032	295219	3.34	5.80	0.43	0.32	6	5
43567	322710	2.78	5.81	0.56	0.19	5	4
47027	350726	2.79	5.83	0.56	0.20	5	4
51542	387570	2.78	5.81	0.56	0.19	5	4
55984	423919	3.39	5.82	0.42	0.33	6	5
61138	465192	3.44	5.83	0.41	0.34	6	5
66622	510201	3.52	5.85	0.40	0.36	6	5
71627	551151	3.48	5.85	0.40	0.35	5	5

Nodes	Edges	PL Exp	Eff. Diam	α	β	Dim_G	Dim_E
77093	594813	3.51	5.85	0.40	0.36	7	–
82869	640416	3.48	5.84	0.40	0.36	7	–
88870	688431	3.46	5.84	0.41	0.36	7	–
95073	738405	3.44	5.85	0.41	0.35	7	–
102902	802009	3.42	5.86	0.41	0.35	7	–
111651	874290	3.33	5.86	0.43	0.34	6	–
122320	963418	3.34	5.85	0.43	0.34	6	–
132572	1051233	3.30	5.83	0.43	0.34	6	–
142230	1131895	3.28	5.87	0.44	0.33	5	–
152676	1219866	3.11	5.85	0.47	0.30	6	–
164333	1322238	3.11	5.86	0.47	0.30	6	–
177058	1436964	3.13	5.85	0.47	0.30	6	–
193345	1582671	3.11	5.85	0.47	0.30	6	–
209628	1732864	3.10	5.83	0.48	0.30	6	–
226563	1886060	3.11	5.85	0.47	0.30	6	–
245398	2058727	3.11	5.85	0.47	0.30	6	–
354977	3184846	3.06	5.78	0.49	0.29	6	–
397649	3675170	3.05	5.77	0.49	0.29	6	–
441553	4214245	3.00	5.75	0.50	0.27	5	–
489997	4827461	2.96	5.71	0.51	0.27	5	–

Table of log-graphlets counts of the LinkedIn networks

Nodes	Edges	$\tilde{G}_1$	$\tilde{G}_2$	$\tilde{G}_3$	$\tilde{G}_4$	$\tilde{G}_5$	$\tilde{G}_6$	$\tilde{G}_7$	$\tilde{G}_8$
578	3140	10.678	8.080	14.270	14.010	12.623	9.442	10.268	8.009
650	3596	11.307	8.431	14.360	14.339	12.850	9.630	10.333	8.593
709	4032	10.984	8.191	14.744	14.447	12.969	9.860	10.536	8.466
774	4448	11.127	8.343	14.544	14.567	12.975	9.870	10.482	8.281
916	5300	11.545	8.548	15.146	15.140	13.552	10.558	10.973	8.324
1012	5983	11.679	8.627	15.359	15.352	13.729	10.604	11.147	9.139
1158	6955	11.975	8.907	15.813	15.704	14.067	11.013	11.402	8.895
1312	7957	12.037	8.987	16.088	15.968	14.128	11.117	11.306	8.937
1512	9287	11.981	8.910	16.181	16.124	14.272	11.245	11.480	9.044
1800	11366	12.312	9.220	16.612	16.586	14.704	11.626	11.908	9.624
2259	14643	12.829	9.611	17.055	16.942	14.908	11.793	12.065	9.777
2643	17795	12.950	9.818	17.430	17.355	15.410	12.197	12.603	10.141
2972	20465	13.370	10.055	17.753	17.616	15.654	12.515	12.816	10.289
3389	23432	13.400	10.158	18.100	17.857	15.849	12.669	12.935	10.441
3817	26656	13.637	10.429	18.340	18.168	16.176	12.856	13.198	10.994
4158	29086	13.756	10.457	18.462	18.271	16.198	12.879	13.258	10.979
4508	31557	13.595	10.275	18.466	18.351	16.277	13.087	13.277	10.947
4898	34273	13.938	10.669	18.724	18.549	16.476	13.110	13.424	11.171
5288	37173	13.934	10.588	18.907	18.709	16.554	13.301	13.486	11.050
5728	40550	14.019	10.704	18.863	18.782	16.633	13.360	13.633	11.138
6216	44091	14.151	10.861	19.020	18.906	16.768	13.440	13.950	11.909
6719	47813	14.166	10.793	19.205	19.043	16.876	13.490	13.894	11.573
7230	51600	14.211	10.757	19.294	19.138	16.985	13.604	14.056	11.973
7892	56238	14.425	11.036	19.416	19.290	17.054	13.675	14.063	11.819
8551	60985	14.603	11.195	19.590	19.467	17.200	13.877	14.183	12.030
9572	68113	14.601	11.177	19.731	19.616	17.340	14.060	14.324	12.466
10444	74215	14.714	11.250	19.884	19.776	17.488	14.081	14.538	12.177
11222	79805	14.846	11.323	20.062	19.938	17.660	14.307	14.618	12.336
12210	87129	14.859	11.328	20.262	20.071	17.785	14.374	14.815	12.742
13043	93158	14.902	11.391	20.347	20.150	17.842	14.340	14.886	12.793

Nodes	Edges	$\tilde{G}_1$	$\tilde{G}_2$	$\tilde{G}_3$	$\tilde{G}_4$	$\tilde{G}_5$	$\tilde{G}_6$	$\tilde{G}_7$	$\tilde{G}_8$
14173	101279	15.075	11.475	20.485	20.336	17.975	14.590	14.979	12.737
15246	109186	15.192	11.585	20.530	20.391	17.970	14.588	14.910	12.745
16499	118284	15.287	11.706	20.780	20.561	18.211	14.738	15.147	12.942
17816	127765	15.348	11.718	20.896	20.700	18.365	14.808	15.328	13.113
19522	140191	15.407	11.786	20.997	20.886	18.490	15.042	15.476	13.284
21125	151651	15.574	11.892	21.147	21.001	18.584	15.123	15.520	13.240
22665	163457	15.545	11.894	21.324	21.098	18.692	15.193	15.657	13.372
24530	177418	15.695	12.010	21.394	21.229	18.813	15.341	15.775	13.531
26535	191947	15.856	12.175	21.575	21.375	18.919	15.421	15.855	13.797
28760	207848	15.901	12.218	21.773	21.496	19.061	15.557	16.028	13.798
31176	225897	16.062	12.383	21.861	21.642	19.204	15.681	16.232	14.142
33834	246160	16.121	12.431	21.976	21.751	19.328	15.833	16.457	14.196
37010	270718	16.109	12.442	22.189	21.905	19.480	15.996	16.560	14.376
40032	295219	16.308	12.580	22.390	22.087	19.681	16.157	16.758	14.438
43567	322710	16.311	12.661	22.513	22.241	19.883	16.388	17.021	14.667
47027	350726	16.447	12.772	22.910	22.449	20.115	16.625	17.254	14.909
51542	387570	16.643	12.907	23.111	22.622	20.307	16.800	17.468	15.314
55984	423919	16.672	13.042	23.136	22.703	20.462	16.986	17.686	15.354
61138	465192	16.785	13.091	23.437	22.882	20.627	17.095	17.831	15.562
66622	510201	16.909	13.252	23.495	23.050	20.757	17.263	17.995	15.727
71627	551151	17.021	13.327	23.814	23.221	20.953	17.420	18.241	15.881
77093	594813	17.326	13.518	24.178	23.393	21.177	17.532	18.447	16.081
82869	640416	17.171	13.494	24.223	23.509	21.307	17.627	18.560	16.126
88870	688431	17.360	13.533	24.481	23.671	21.411	17.763	18.696	16.325
95073	738405	17.335	13.648	24.491	23.778	21.467	17.780	18.650	16.217
102902	802009	17.494	13.791	24.777	24.000	21.704	17.954	18.901	16.598
111651	874290	17.561	13.771	24.827	24.137	21.795	18.075	18.979	16.630
122320	963418	17.725	13.903	25.348	24.403	22.081	18.252	19.307	16.934
132572	1051233	17.969	14.055	25.645	24.607	22.280	18.363	19.539	17.232
142230	1131895	18.002	14.160	25.824	24.723	22.382	18.438	19.612	17.297
152676	1219866	18.035	14.195	26.271	24.898	22.631	18.627	19.866	17.313
164333	1322238	18.129	14.247	26.387	25.058	22.757	18.709	19.881	17.430
177058	1436964	18.531	14.481	26.716	25.250	22.942	18.814	20.034	17.458
193345	1582671	18.459	14.531	26.974	25.471	23.162	19.039	20.179	17.742
209628	1732864	18.681	14.664	27.251	25.644	23.265	19.148	20.219	17.881
226563	1886060	18.917	14.762	27.405	25.809	23.464	19.330	20.427	18.021
245398	2058727	18.849	14.867	27.594	25.941	23.604	19.391	20.522	18.135
354977	3184846	19.350	15.411	28.372	26.869	24.554	20.246	21.400	19.064
397649	3675170	19.705	15.624	28.791	27.215	24.904	20.512	21.733	19.452
441553	4214245	20.009	16.003	29.337	27.588	25.406	20.909	22.201	19.979
489997	4827461	20.415	16.301	29.879	28.002	25.887	21.298	22.579	20.384

References and Notes

[1] M. Bayati, J.H. Kim, A. Saberi, Algorithmica (2010) 58: 860910.

[2] Gleich, `https://www.github.com/dgleich/bisquik`

[3] A. Bonato, J. Janssen, P. Prałat. Internet Mathematics **8** 2012, 2–28.

[4] S. Janson, T. Łuczak, A. Rucinski, *Random graphs*, Wiley-Intersci. Ser. Discrete Math. Optim. (2000).
